# Successful treatment of recurrent benign fibrous histiocytoma by complete surgical removal and appropriate ocular surface reconstruction: A case report

**DOI:** 10.1097/MD.0000000000022941

**Published:** 2020-10-30

**Authors:** Haixu Wang, Shuang Zhang, Jing Hong

**Affiliations:** Department of Ophthalmology, Peking University Third Hospital, Key Laboratory of Vision Loss and Restoration, Ministry of Education, Beijing, China.

**Keywords:** benign, fibrous histiocytoma, ocular reconstruction, recurrent

## Abstract

**Introduction:**

Ocular benign fibrous histiocytoma can involve corneoscleral limbus and adjacent cornea and usually has a good prognosis after surgical removal. Despite the low recurrence rate, we reported a rare case of ocular benign fibrous histiocytoma with twice recurrences after excision.

**Patient concerns:**

A 12-year-old Chinese girl presented with two painless progressively enlarging masses in the right eye for 6 years. She once had the lesions excised 1 year ago. However, the primary lesions relapsed again.

**Diagnosis:**

Histopathologic and immunohistochemical examinations of the excised samples supported the diagnosis of benign fibrous histiocytomas of the corneoscleral limbus.

**Interventions:**

The patient underwent mass resection with limbal stem cell transplantation and amniotic membrane transplantation at first. As for the tumors’ second recurrence, we performed extended excision combined with lamellar keratoplasty and amniotic membrane implantation.

**Outcomes:**

The corneal graft remained clear with no sign of tumor recurrence 3 years after the second surgery.

**Conclusion:**

Complete surgical resection with tumor-free margins is critical to reduce the recurrence of benign fibrous histiocytoma and appropriate ocular surface reconstruction is necessary to remedy tissue defect and maintain epithelial integrity.

## 1 Introduction

Fibrous histiocytoma (FH) is among the most common primary mesenchymal tumors of the orbit.^[1]^ They are usually classified histologically as either benign or malignant, and the former is more common.^[1]^ The mainstay treatment of benign FH is complete resection with tumor-free margins^[2]^ and it seems that the recurrence rate is low according to previous publications.^[3–12]^ Since the tumor can grow from peripheral conjunctiva to corneoscleral limbus and even penetrate deeply into corneal stroma,^[7]^ complete mass excision might result in large-scale stromal defect and limbal stem cell defect, in which situation appropriate ocular reconstruction such as lamellar keratoplasty, limbal stem cell transplantation and amniotic membrane transplantation is necessary. Here we reported a rare case of benign fibrous histiocytoma with twice recurrences after excision and finally controlled by a third surgery.

## 2 Case report

**Figure 1:**
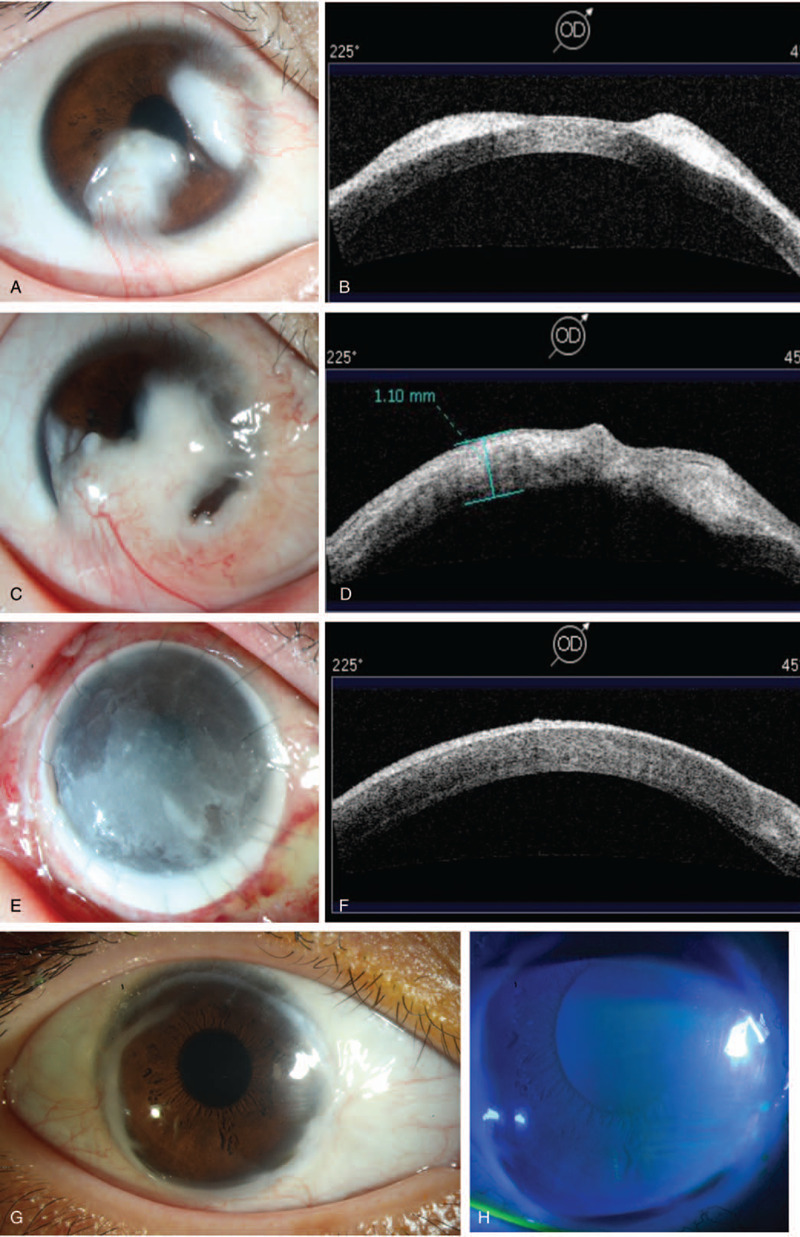
The appearance during tumor's clinical treatment course. (A) Original view of the lesions by slit-lamp examination: Two masses arising from the corneoscleral limbus that is moderately vascular and had a similar appearance to a dermoid cyst. (B) Anterior segment optical coherence tomography (AS-OCT) showed the two lesions (45°–235°) were well-defined with hyperintensity and invaded anterior stroma. (C) Six months after the first resection, the 2 primary lesions relapsed and grew progressively to combine with each other, occluding the pupillary area. (D) The recurrent tumors invaded deeply into the posterior stroma with a poorly defined boundary but not protruded the DM by AS-OCT (45°–235°). (E) Postoperative 1-week, slit-lamp examination showed the large lamellar graft with corneoscleral rims (11.5 mm in diameter), covered by the residual amniotic membrane. (F) The graft attached well to the posterior stroma with high reflective amniotic membrane by AS-OCT (45°–235°). (G) The graft was still clear with circular epithelial haze peripherally 3 yr after the second surgery. (H) The corneal epithelium was intact by fluorescein staining postoperative 3 yr.

A 12-year-old healthy Chinese girl presented with two enlarging masses in her right eye for 6 years without any ocular symptoms. The lesions had been excised at another hospital 1 year ago but relapsed 4 months after excision. The slit lamp examination showed two whitish flat-domed conjunctival masses invaded the corneoscleral limbus and adjacent cornea with multiple superficial conjunctival vessels passing over the base of the lesions (Fig. 1A). According to the anterior segment optical coherence tomography (AS-OCT), the lesions were highly reflective with clear margin and broke through Bowman membrane, invading the anterior stroma (Descemet membrane [DM]) (Fig. 1B). Since the inferior lesion covered the pupil area partially, visual acuity (VA) of her right eye was 20/100. She subsequently underwent surgical removal of the two masses, together with limbal stem cell transplantation (ranging from 1 to 7 o’clock) and amniotic membrane transplantation at our hospital. The excised masses were sent for histopathologic examination.

**Figure 2:**
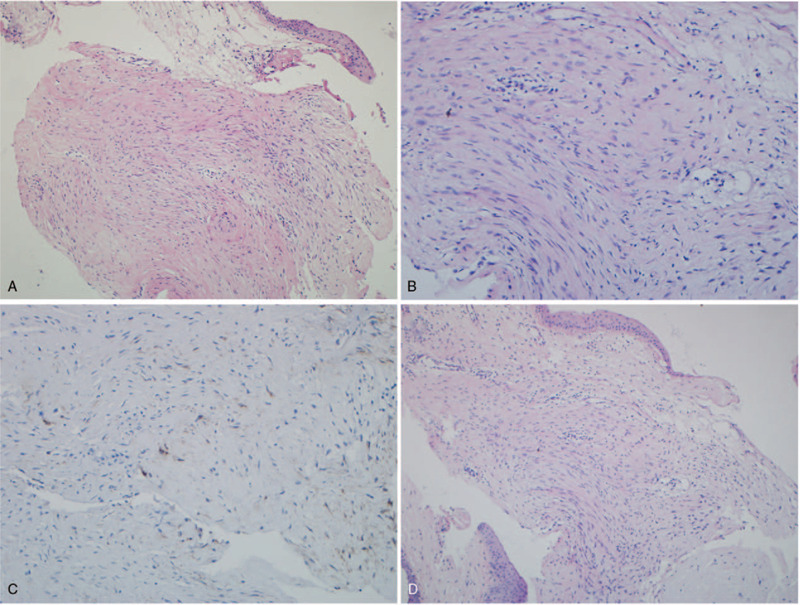
Histopathological examination of the excised masses. (A) Light microscopic view of surgical specimen revealed the tumors were composed of interweaving fascicles of spindle cells in a fibrous matrix and invaded adjacent corneal stroma. Hematoxylin-eosin (H&E), original magnification ×10. (B) A fascicular growth pattern with a large amount of collagen. H&E stain, original magnification ×20. (C) Smooth muscle actin (SMA) stain positive for the spindle cells of the lesion, original magnification ×20. (D) The recurrent tumors from the second surgery were characteristic of a hypercellular nodule including spindle cells with peripheral lymphocyte infiltration; Tumor-free margin was noted with normal corneal stroma. H&E stain, original magnification ×10.

Histopathological examination disclosed a fine collagenous network composed of spindle-shaped cells without nuclear atypia. Histiocytes, small lymphocytes and scattered multinucleated giant cells were also noted. The tumors partially penetrated into the deep aspect of the corneal stromal fibrous layers (Fig. 2A). Individual cells were arranged in a fascicular pattern with a hyperchromatic nucleus, acidophilic cytoplasm and collagen deposition (Fig. 2B). Immunohistochemical analysis demonstrated that the spindle cells of the lesion were positive for smooth muscle actin (Fig. 2C), negative for soluble protein-100, antigen Ki67, and alcian blue. Given all the above pathological features, ocular benign fibrous histiocytoma was diagnosed.

However, the tumors relapsed 2 months later; The recurrent lesions grew progressively to occlude the central cornea with clear dilated superficial vessels in the inferior area 6 months after the last surgery (Fig. 1C). The tumors invaded deeply into the posterior stroma without penetration of DM by AS-OCT (Fig. 1D). With all the above factors considered, we finally decided to perform another surgery on her. For this time, the lesions were excised completely with 1 mm safety margins; The extensive corneal stroma defect was compensated by a lamellar corneal graft with corneoscleral rims, as large as 11.5 mm in diameter (Fig. 1E); Besides, amniotic membrane transplantation was also performed to cover the graft for prevention of epithelial defect. As AS-OCT revealed, the graft attached well to the residual posterior stromal layer with the amniotic membrane over the surface postoperative one week (Fig. 1F). Histopathological examination of the excised mass also revealed recurrent benign fibrous histiocytoma. The lesion showed a nodular pattern with lymphocyte infiltration peripherally (Fig. 2D). The second surgery turned out to be successful. After 3-year follow-up, her cornea remained clear and there was no sign of tumor recurrence (Fig. 1G); The corneal epithelium was intact by fluorescein staining (Fig. 1H). Her VA recovered to 20/33 in her right eye at the final visit.

## 3 Discussion

Benign fibrous histiocytoma of the corneoscleral limbus behaves similarly to those occurring elsewhere on the body, with an excellent prognosis. A remnant tumor is associated with local recurrence.^[13]^ Since the tumors can grow progressively into large masses, sometimes they can invade corneoscleral limbus, deeper corneal stroma and even protrude into anterior chamber.^[14]^ In that case, it is quite a dilemma for surgeons to decide whether to achieve enough tumor-free margins for the cost of large-scale tissue defect; Tissue defect including corneal stroma or limbal stem cell requires keratoplasty, which means more complicated postoperative management and increased patient's burden. In this case, we managed to excised the tumor completely without too much damage for the first time since the tumors were limited to anterior corneal stroma in dept by AS-OCT and the patient was quite young. However, it was not enough and the lesions relapsed in short time. The recurrent lesions appeared to grow more rapidly and presented more aggressive clinical features. In the second recurrence, the tumor had a diffuse invasion of the deep corneal stroma, poorly cleared margins and extensively infiltrated the surrounding tissues. With all the above factors, we tried our best to achieve complete mass resection with 1 mm safety margins. In order to remain the DM and endothelium, we did not pursue more tumor-free margins given the fact of better prognosis of lamellar keratoplasty than penetrating keratoplasty. We chose to cover the corneal defect by a large lamellar graft with corneoscleral rims for compensation of most limbal stem cell deficiency and better postoperative VA. Besides, we also performed amniotic membrane transplantation at the same time to facilitate ocular surface recovery. The second surgery turned out to be very successful. Therefore, complete excision with tumor-free margins should be managed and appropriate ocular reconstruction was also important to maintain ocular surface's stability especially for large mass resection.

Malignant fibrous histiocytoma characteristically appears in later life. In contrast, benign corneoscleral fibrous histiocytomas can develop at any age.^[8]^ A definitive diagnosis is confirmed by histopathologic evaluation, which morphologically shows a storiform pattern composed of short, twisting fascicles of cells^[4,15]^ Unlike malignant fibrous histiocytomas, no cellular atypia, nuclear pleomorphism or mitotic activity is observed in benign cases.^[5]^ However, benign fibrous histiocytoma can show an infiltrative growth pattern and local recurrence, similar to malignant fibrous histiocytoma.^[13]^ The recurrent lesions can appear within a few weeks after a conservative resection and grow rapidly. To avoid it, complete surgical resection with tumor-free margins followed is critical, as is closely follow-up in the postoperative period.

## Acknowledgments

Many people have offered us valuable help in our article writing. We would like to give our sincere gratitude to Prof. Hong Jing, our supervisor with her extraordinary patience and consistent encouragements.

## Author contributions

**Supervision:** Jing Hong.

**Writing – original draft:** Haixu Wang, Shuang Zhang.
